# Supplementary material for: Altered microRNA Transcriptome in Cultured Human Liver Cells upon Infection with Ebola Virus
Source: Int J Mol Sci. 2021 Apr 6;22(7):3792. doi: 10.3390/ijms22073792 (PMC8038836; doi:10.3390/ijms22073792)
Supplement: Supplementary file 1 [file ijms-22-03792-s001.zip › Supplementary_File/C_ GO_Analysis_Results/16-30nt_go_Reston-24h-Huh7_vs_Control-24h-Huh7_down.mature_mirna_targets/MF_Pvalue_tree.pdf]

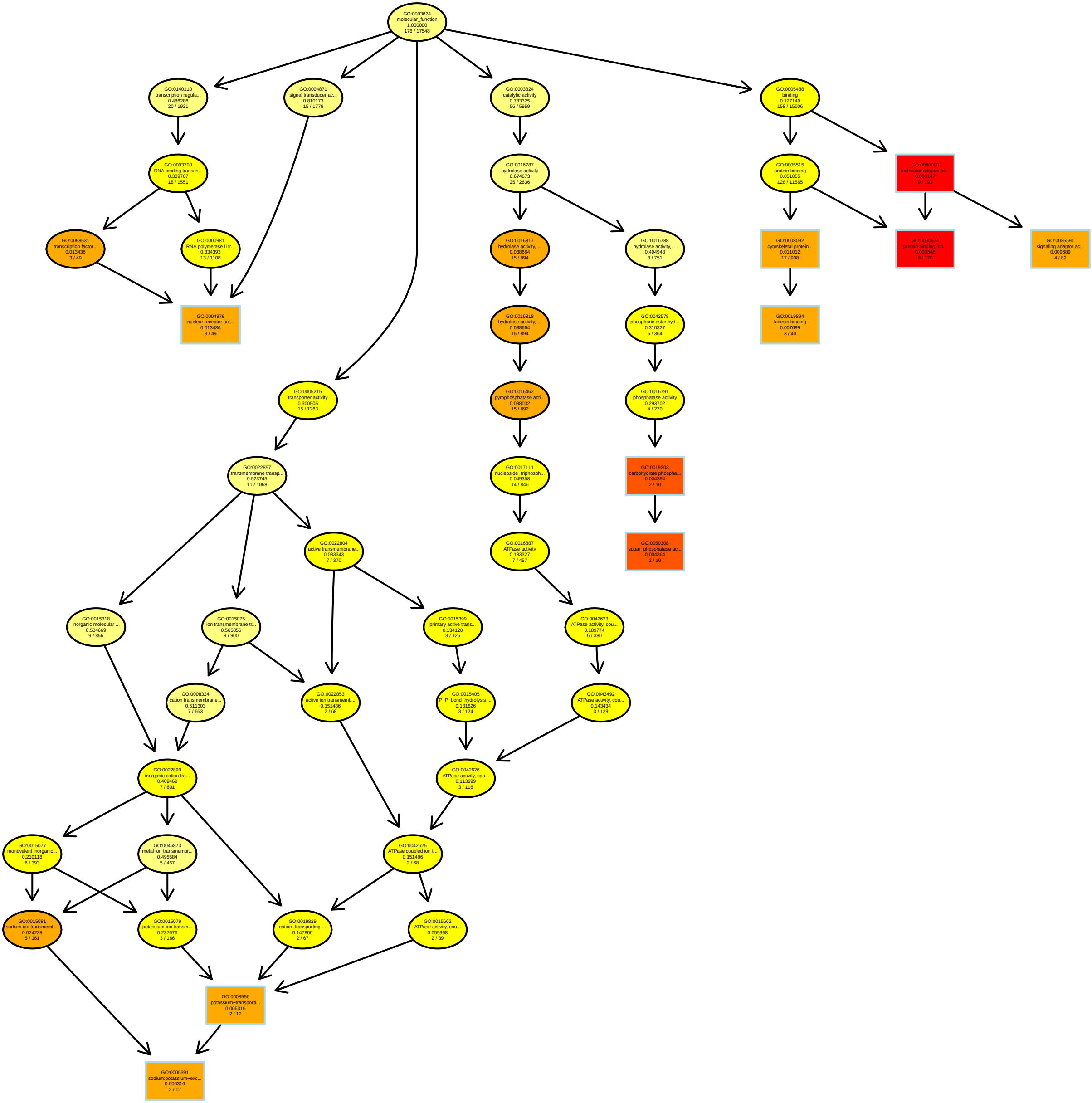

GO:0003674 molecular_function 1.000000 178 / 17548
GO:0140110 transcription regula... 0.486286 20 / 1921
GO:0004871 signal transducer ac... 0.810173 15 / 1779
GO:0003824 catalytic activity 0.783325 56 / 5959
GO:0005488 binding 0.127149 158 / 15006
GO:0003700 DNA binding transcri... 0.309707 18 / 1551
GO:0016787 hydrolase activity 0.674673 25 / 2636
GO:0005515 protein binding 0.051055 128 / 11565
GO:0060090 molecular adaptor ac... 0.000147 9 / 191
GO:0098531 transcription factor... 0.013436 3 / 49
GO:0000981 RNA polymerase II tr... 0.334393 13 / 1108
GO:0016817 hydrolase activity, ... 0.038664 15 / 894
GO:0016788 hydrolase activity, ... 0.494948 8 / 751
GO:0008092 cytoskeletal protein... 0.011012 17 / 908
GO:0030674 protein binding, bri... 0.000348 6 / 170
GO:0035591 signaling adaptor ac... 0.009689 4 / 82
GO:0004879 nuclear receptor act... 0.013436 3 / 49
GO:0016818 hydrolase activity, ... 0.038664 15 / 894
GO:0042578 phosphoric ester hyd... 0.310327 5 / 364
GO:0019894 kinesin binding 0.007699 3 / 40
GO:0005215 transporter activity 0.300505 15 / 1263
GO:0016462 pyrophosphatase acti... 0.038032 15 / 892
GO:0016791 phosphatase activity 0.293702 4 / 270
GO:0022857 transmembrane transp... 0.523745 11 / 1068
GO:0017111 nucleoside−triphosph... 0.049358 14 / 846
GO:0019203 carbohydrate phospha... 0.004364 2 / 10
GO:0022804 active transmembrane... 0.083343 7 / 370
GO:0016887 ATPase activity 0.183327 7 / 457
GO:0050308 sugar−phosphatase ac... 0.004364 2 / 10
GO:0015318 inorganic molecular ... 0.504669 9 / 856
GO:0015075 ion transmembrane tr... 0.565856 9 / 900
GO:0015399 primary active trans... 0.134120 3 / 125
GO:0042623 ATPase activity, cou... 0.189774 6 / 380
GO:0008324 cation transmembrane... 0.511303 7 / 663
GO:0022853 active ion transmemb... 0.151486 2 / 68
GO:0015405 P−P−bond−hydrolysis−... 0.131826 3 / 124
GO:0043492 ATPase activity, cou... 0.143434 3 / 129
GO:0022890 inorganic cation tra... 0.409469 7 / 601
GO:0042626 ATPase activity, cou... 0.113999 3 / 116
GO:0015077 monovalent inorganic... 0.210118 6 / 393
GO:0046873 metal ion transmembr... 0.495584 5 / 457
GO:0042625 ATPase coupled ion t... 0.151486 2 / 68
GO:0015081 sodium ion transmemb... 0.024238 5 / 161
GO:0015079 potassium ion transm... 0.237676 3 / 166
GO:0019829 cation−transporting ... 0.147966 2 / 67
GO:0015662 ATPase activity, cou... 0.059368 2 / 39
GO:0008556 potassium−transporti... 0.006316 2 / 12
GO:0005391 sodium:potassium−exc... 0.006316 2 / 12
